# Construction of Highly Conductive Cross-Linked Polybenzimidazole-Based Networks for High-Temperature Proton Exchange Membrane Fuel Cells

**DOI:** 10.3390/ma16051932

**Published:** 2023-02-26

**Authors:** Tianyang Li, Jiayu Yang, Qingxin Chen, Hui Zhang, Peng Wang, Wei Hu, Baijun Liu

**Affiliations:** 1 Key Laboratory of High Performance Plastics of the Ministry of Education, National & Local Joint Engineering Laboratory for Synthesis Technology of High Performance Polymer, College of Chemistry, Jilin University, 2699 Qianjin Street, Changchun 130012, China; 2 Key Laboratory of Polyoxometalate Science of the Ministry of Education, Faculty of Chemistry, Northeast Normal University, 5268 Renmin Street, Changchun 130024, China

**Keywords:** high-temperature proton exchange membranes, fuel cells, polybenzimidazole, cross-linking, proton conductivity

## Abstract

High-temperature proton exchange membrane fuel cells (HT-PEMFCs) are of great interest to researchers in industry and academia because of their wide range of applications. This review lists some creative cross-linked polybenzimidazole-based membranes that have been prepared in recent years. Based on the investigation into their chemical structure, the properties of cross-linked polybenzimidazole-based membranes and the prospect of their future applications are discussed. The focus is on the construction of cross-linked structure of various types of polybenzimidazole-based membranes and their effect on proton conductivity. This review expresses the outlook and good expectation of the future direction of cross-linked polybenzimidazole membranes.

## 1. Introduction

The exhaust gases produced by the combustion of fossil fuels are high in harmful gases, causing serious environmental pollution faced in recent years, which has a great impact on the daily work and life of humans. Decarbonization has become the voice of people all over the world. Electrochemistry is one of the most effective ways to decarbonize. Hydrogen fuel cells are currently considered as an effective technological solution to achieve energy security, reliability, efficiency and sustainability [1,2,3]. Fuel cells are power generation devices that directly convert chemical energy stored in fuels into electrical energy and their energy conversion efficiency can be very high [4,5]. Platinum and platinum group metals have been chosen as catalysts for PEMFCs because of the acidic operating environment of PEMFCs, which corrodes metals. To date, the most advanced catalysts are Pt or Pt alloy nanoparticles, typically dispersed on a porous carbon carrier. Because the reaction product of fuel cells is only water, fuel cells can be used as a green energy resource [6,7]. Compared with anion exchange membrane fuel cells (AEMFCs) and some other types of fuel cells [8,9,10,11], proton exchange membrane fuel cells (PEMFCs) have attracted wide attention and made great research progress because of their very high commercial value due to their small size, high energy conversion rate, high proton conductivity, high output power density, good reliability and no pollution [12,13]. HT-PEMFCs are used in stationary and portable applications. For example, methanol-fueled HT-PEMFCs are used as replacements for generators and range extensions for electric vehicles. Typically, HT-PEMFCs are used in conjunction with batteries for hybrid operation. Natural-gas-fueled HT-PEMFCs are also used for cogeneration applications in buildings. HT-PEMFCs have the potential to revolutionize the heavy-duty transportation industry: allowing ships to run on renewable methanol, ammonia, aircraft to run on DME or hydrogen, and off-grid generators to be easily transported to remote areas using low or zero carbon fuels. HT-PEMFCs have the potential to revolutionize the heavy-duty transportation industry: allowing ships to run on renewable methanol or ammonia, aircraft to run on DME or hydrogen, and off-grid generators to be easily transported to remote locations using low or zero carbon fuels. PEMFCs can be divided into low-temperature proton exchange membrane fuel cells (LT-PEMFCs) and high-temperature proton exchange membrane fuel cells (HT-PEMFCs) according to their operating temperature and humidity. In contrast to LT-PEMFCs, HT-PEMFCs may generally possess the following characteristics: (1) improved electrochemical kinetics, (2) simplified water/thermal management system, (3) a reduced probability of CO-poisoned anodes and (4) lower costs [14,15]. As one core component of the AEMFCs and PEMFCs, polymeric membranes, such as anion exchange membranes (AEMs) and proton exchange membranes (PEMs), play the role of anion or proton conductions, fuel and oxidant separation, and their properties largely determine the performance of fuel cells [16,17,18,19]. Therefore, the research on functional membranes for AEMFCs and PEMFCs has attracted much attention. Some examples of polymers with different functional groups (e.g., quaternary ammonium, sulfonic acid, imidazole and so on) mainly from our research group for membranes in AEMFCs, LT-PEMFCs and HT-PEMFCs are summarized in Table 1. The table summarizes the main properties of the membranes for the three cells, innovatively. It is found that, in general, HT-PEMFCs have higher temperature range, proton conductivity and peak power density. Therefore, HT-PEMFCs have been the most discussed hotspots within the industry.

**Figure 1:**
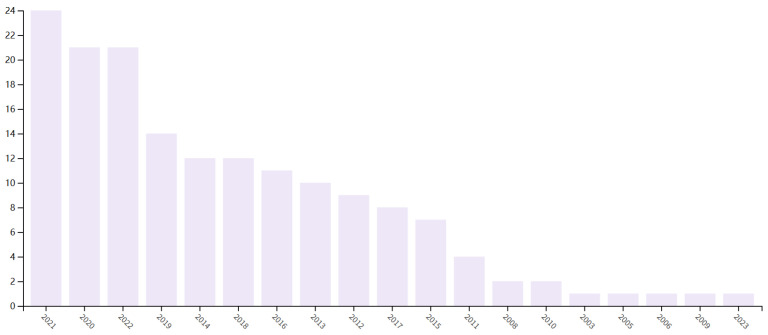
The bibliometric analysis of cross-linked polybenzimidazole for high-temperature proton exchange membrane fuel cells.

This review also carries out a bibliometric analysis of cross-linked polybenzimidazole for high-temperature proton exchange membrane fuel cells. According to the number of SCI core journals published in this field in recent years, Figure 1 is obtained. As can be seen from the figure, the subject has become a hot topic of current research in recent years. The number of journals published in recent years is increasing and the quality is getting higher. Proton exchange membranes are the core material of fuel cells, and the performance of proton exchange membranes will directly affect the industrialization process of fuel cells and is one of the key factors to achieving large-scale applications. In order to realize the practical application and industrialization of fuel cells, a lot of research has been conducted on the manufacturing process and material modification of proton exchange membranes. At present, the further improvement of the durability, lifetime and working performance of proton exchange membranes is still an important task facing the industrialization of proton exchange membrane fuel cells. The fuel cell market is still an emerging market and has not reached a large scale worldwide. Driven by the market demand for fuel cells, proton exchange membranes will certainly gain further development. It is believed that higher performance and lower cost proton exchange membrane products will be available soon, which will vigorously promote the development of fuel cell technology and its industrial application. The technology readiness level (TRL) is a metric that describes the degree to which a technology has reached commercialization. There are nine levels of TRL. Nowadays, the TRL of PEM is 6–8. In recent years, the commercialization of PEM has been getting closer and closer, and countries have started to develop PEM research efforts vigorously.

## 2. Polybenzimidazole-Based Membranes

**Figure 2:**
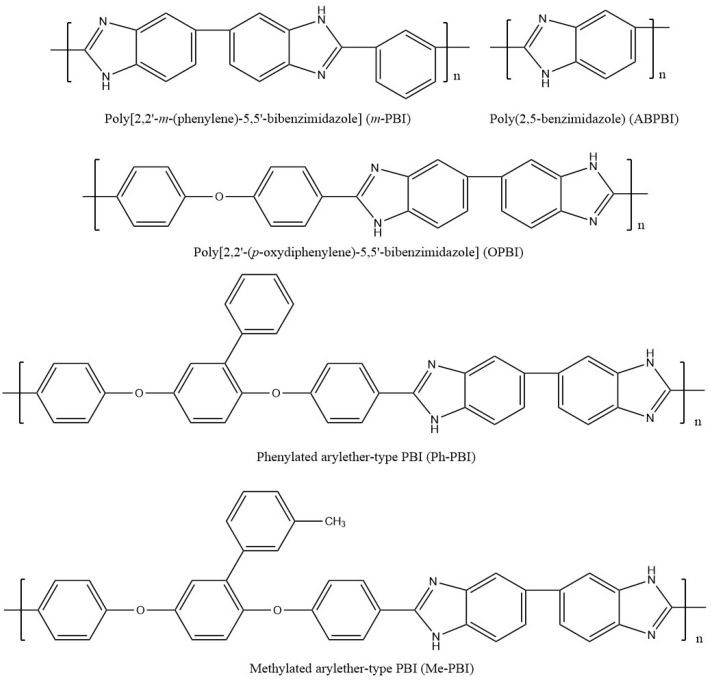
Chemical structure of some PBIs.

Polybenzimidazoles (PBIs) are a class of heterocyclic polymers containing benzimidazole groups in repeating units, and they usually have excellent thermal stability, mechanical strength and chemical resistance [36,37,38]. Generally, their syntheses are difficult because of their unique rigid main chain structure containing benzimidazole moieties [39]. The structural formulas of some PBI polymers are shown in Figure 2. The solution polymerization method is the main method for the preparation of PBIs synthesized from tetraamines and diacids at 170–190 ${}^{\circ }$C in the laboratory at present [31,40]. In 1995, M. Litt et al. [41] at Case Western Reserve University first doped phosphoric acid (PA) into PBI, and the resulting phosphoric-acid-doped polybenzimidazole (PA-PBI) membrane exhibited high proton conductivity, good mechanical properties and high thermal stability at a high temperature above 100 ${}^{\circ }$C and low humidity conditions. Bjerrum’s group [42] pointed out that phosphoric-acid-doped polybenzimidazole membranes appear to be the most successful system in the field of proton exchange membranes to date. To date, PA-PBI type proton exchange membranes have become a hot topic in the research field of high-temperature proton exchange membranes due to their excellent properties [43,44,45] and they are the most promising candidate membranes for HT-PEMFCs [46,47]. Currently, the catalysts for HT-PEMFCs are mainly made of PGM materials, while PGM-free catalysts have been reported in recent years, but their overall performance is still inferior to that of PGM catalysts. PBI membranes such as Celazole^®^ PBI and Celtec^®^ have been successfully commercialized, with a glass transition temperature of 427 ${}^{\circ }$C compared to the 120–140 ${}^{\circ }$C glass transition temperature of common Nafion membranes, which also means their excellent thermal stability and progressive application in fuel cells under high temperature and high pressure conditions. Celazole^®^ PBI is a product of PBI USA, which was the first to commercialize PBI membranes in 2000, and its main products are Celazole^®^ U series and T series polymers. In addition, BASF (Ludwigshafen, Germany) also commercialized PBI membranes in 2003, and its Celtec^®^ PBI membranes are mainly for high-temperature fuel cells, and it has commercialized Celtec^®^-P membrane electrodes based on PBI membranes. The main limitations of high-temperature proton exchange membrane fuel cells are the bipolar plate corrosion phenomenon and the maintenance of high operating temperatures, but interest in them in the fuel cell field continues to grow. For high-temperature proton exchange membrane fuel cells, polybenzimidazole membranes are uniquely suited and are the preferred choice for this system.

Savinell et al. [9] suggested that proton transfer in PA-PBI membranes would occur along different proton donor–acceptor chains. Therefore, the proton conductivity of PA-PBI membrane depends on the doping level of phosphoric acid in the membrane [48]. The proton conductivity increases with the increase of acid doping level (ADL) [49], but the mechanical strength of the membrane will decrease significantly with the increase of ADL due to the plasticizing effect of PA on PBI membrane, especially under high temperature conditions [50]. This will greatly affect the cell properties of the membranes. For instance, after doping with PA, the tensile strength of the m-PBI membrane decreased from 42.3 MPa to 3.4 MPa [51]. Therefore, it is significant to improve the maintenance of excellent mechanical properties at high phosphoric acid doping levels [52]. PA-PBI type proton exchange membrane is an important issue to be solved in the research [53]. It is also a prerequisite for the production of fuel cells with high performance and long life. The loss of phosphoric acid from the PBI membrane during cell operation is also an important factor, which affects the properties and lifetime of the cell [54]. Bjerrum et al. [55] suggested that the proton conductivity of the PA-PBI membrane decreases with the loss of phosphoric acid, which in turn affects the overall properties of the cell.

In order to achieve a higher proton conductivity, a better mechanical strength and a more stable phosphoric acid retention, researchers have used various methods to modify PBI membranes [56,57], including the synthesis of PBI polymers with novel backbone structures, chemical grafting or cross-linking modification of PBIs, and the preparation of inorganically doped composite membranes, etc. In our previous work [58], some novel backbone structures of PBIs (e.g., Ph-PBI and Me-PBI) were synthesized and investigated for HT-PEMs. In contrast, flexible ether linkages and asymmetric bulky pendants are both important for improving some properties. Our group [34] also reported that grafted PBIs were prepared by an effective N-substitution method without catalyst. The g-PBI-20 membrane exhibited a conductivity of 0.212 S·cm${}^{-1}$ at 200 ${}^{\circ }$C and a peak power density of 443 mW·cm${}^{-2}$. A series of PBI-based composite membranes were prepared by adding SPAEK as a compatibilizer to Ph-PBI and SPOSS was doped into the system by the sol-gel method [31]. The addition of sulfonic acid groups could substantially improve the proton conductivity and SPOSS has a cage-like structure, which could improve the phosphoric acid adsorption level and retention ability. As a result, the highest proton conductivity of the composite membrane was 0.126 S·cm${}^{-1}$ at 200 ${}^{\circ }$C and the maximum power density was 300 mW·cm${}^{-2}$ at 160 ${}^{\circ }$C. It was reported that the OPBI membrane with hyperbranched structure was prepared [32]. Due to the introduction of the hyperbranched structure, this type of membrane has excellent dimensional stability and mechanical strength, which is 233% higher than the mechanical strength of the pristine OPBI membrane. Wang’s group [59] designed and synthesized a series of highly branched polybenzimidazoles. The ADL of the branched PBI membranes with a 9% degree of branching was 10.5, which was a great improvement compared with PBI. Bjerrum et al. [60] pointed out that the cross-linked PBI had more advantages than other PBIs. It was reported by us that a series of cross-linked membranes were prepared using Allyl-SPAEK as the cross-linker and OPBI by UV irradiation, constructing a PBI/Allyl-SPAEK semi-interpenetrating polymer network with strong intermolecular interactions [33]. The maximum tensile strength of the PBI/Allyl-SPAEK-20% blend membrane reached 28.5 MPa, which was 2.16 times higher than that of the pristine OPBI membrane. At 200 ${}^{\circ }$C, the proton conductivity of PBI/Allyl-SPAEK-20% reached 0.206 S·cm${}^{-1}$, which was 38% higher than that of pristine OPBI membrane. Our group prepared a series of alloyed membranes with a special intrinsic “porous” structure by incorporating PIM-1 into OPBI [35]. PIM-1 had a strong anti-plasticizing effect and substantially improved the mechanical properties of the alloy membranes. The alloy membranes exhibited excellent properties, a proton conductivity of 313 S·cm${}^{-1}$ at 200 ${}^{\circ }$C and a peak power density of 438 mW·cm${}^{-2}$ at 160 ${}^{\circ }$C. Cross-linking is the process by which linear or branched polymer chains are covalently bonded to each other to form a network or bulk polymer. This reaction transforms linear or lightly branched macromolecules into a three-dimensional network structure, thereby improving strength, heat resistance, wear resistance, solvent resistance and other properties. Constructing a cross-linked network can substantially enhance the dimensional stability, mechanical properties and plasticization resistance of the membranes. If the proton exchange membrane is not strong enough, it tends to rupture and perforate, leading to the mixing of cathode and anode gases with each other. Cross-linked membranes can be made thinner than other membranes which reduces the internal resistance of the fuel cell. This review focuses on the effect of cross-linking modification of PBI on its properties.

## 3. Cross-Linked Polybenzimidazole Membranes

**Figure 3:**
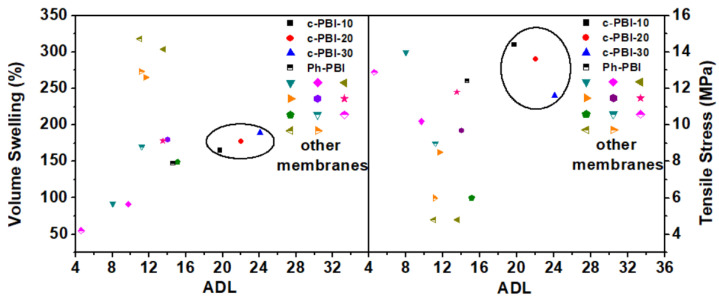
The effects of cross-linking on the mechanical strength and dimensional stability (taken from Ref. [65]).

As illustrated in Figure 3, cross-linking is an effective way to directly enhance the mechanical strength and dimensional stability of polymeric membranes [61,62]. For PA-PBI proton exchange membranes, the plasticizing effect of PA doping on PBI membranes, especially at high ADL, causes significant damage to the mechanical properties of the membranes [63,64]. Cross-linking reaction is a reaction in which two or more molecules are bonded to each other and cross-linked into a network structure of more stable molecules. Cross-linking is divided into chemical cross-linking and physical cross-linking. Chemical cross-linking is generally achieved through polycondensation and polymerization reactions, while physical cross-linking uses radiation such as light and heat to cross-link linear polymers. For examples, thermal cross-linking is a cross-linking method in which a cross-linked network is created by heat treatment at high temperatures. Solvothermal cross-linking is a cross-linking method in which a cross-linked network is formed by heat treatment at low temperatures for a short period of time. Radiation assisted cross-linking is a cross-linking method in which a cross-linked network is created by electron beam (EB) and ultraviolet (UV) radiation at room temperature. After moderate cross-linking of linear polymers, their mechanical strength, elasticity, dimensional stability and solvent resistance are improved. Linear structure (including branched structure) polymers have the characteristics of elasticity, plasticity, solubility in solvents, melting when heated, and less hardness and brittleness due to the presence of independent molecules. The bulk structure polymers have no elasticity and plasticity because no independent macromolecules exist, so they cannot be dissolved and melted, only swollen, and have greater hardness and brittleness. It was demonstrated that cross-linking could reduce the dramatic softening of the membranes doped with phosphoric acid. Therefore, the cross-linked membranes could maintain a higher ADL, leading to a higher proton conductivity. Furthermore, in the Fenton test, the cross-linked membranes had a reduced weight loss rate. The cross-linked PA-PBI membranes are expected to maintain good mechanical properties and durability of the membrane even at high ADLs (Table 2). There are many methods for constructing cross-linked PBI networks [65]: (1) Lewis acid-base reactions via blending basic PBIs with acidic polymers; (2) typical N-substitution reactions of PBIs with cross-linking agents containing halogen or epoxy groups; (3) thermal curing, Friedel–Crafts and Diels–Alder reactions, and other reactive groups. The use of high-temperature proton exchange membranes is less dependent on high-purity hydrogen production and storage which allows the direct use of impure hydrogen from methanol reforming, which can greatly simplify fuel cell systems and increase the reliability of fuel cells. High-temperature proton exchange membrane fuel cells have great potential in the heavy-duty transportion industry, where they can significantly reduce the weight of heavy-duty vehicles. As a branch of the high-temperature proton exchange membrane field, the future of cross-linked polybenzimidazole membranes is very promising and is believed to have great advantages and potential for use in backup power, auxiliary power units and heavy-duty transportation.

In Wang’s work [66], Br-HPP with hyperbranched structure was used as the cross-linker of the system. This cross-linker was characterized by large size and many functional groups, which could form multiple cross-linking sites and provided favorable conditions for subsequent functional group modifications. It could react with the benzimidazole groups to form a cross-linked network via the formation of amide-type bonds. The quaternary ammonium (QA) group, which was strongly basic, was introduced into the polymer, and it could promote the dissociation of PA and established acid–base pairs, so the QOPBI cross-linked membrane had good phosphoric acid adsorption and retention ability. The cross-linked QOPBI membrane had better thermal stability and oxidation resistance. The tensile strength of the PA-doped cross-linked membranes exceeded 20.0 MPa. All QOPBI membranes exhibited better proton conductivity. In the fuel cell test of QOPBI-15 membrane, it showed a peak power density of 260 mW·cm${}^{-2}$.

**Figure 4:**
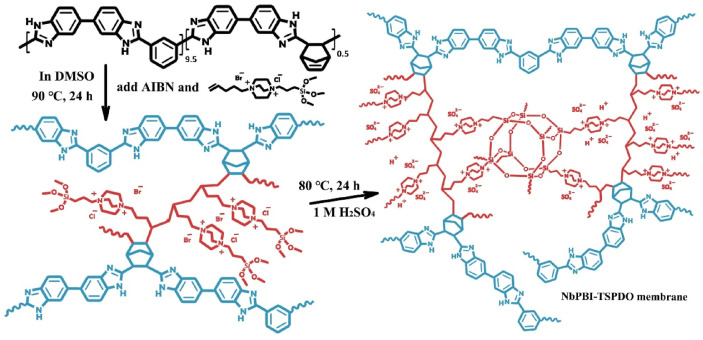
The cross-linked structure of NbPBI-TSPDO (taken from Ref. [67]).

Wang et al. reported that a double cross-linked composite membrane was prepared by cross-linking 1-(3-(trimethoxysilan)propyl)-4-(5-pentenyl)-1,4-diazoniabicyclo-[2.2.2]-octane bromide chloride ([TSPDO]BrCl) with norbornene-type polybenzimidazole (NbPBI) bearing a trimethoxysilan group [67]. The author polymerized the poly (ionic liquid) (PIL) by in situ free radical reactions, which was firstly cross-linked with NbPBI. After that, hydrolysis reaction of the trimethoxysilane groups occurred, forming the second cross-linked network. The Si-O-Si cross-linked network was formed by a sol-gel method during the membrane fabrication process (Figure 4). The NbPBI-TSPDO cross-linked membranes exhibited enhanced stability, mechanical properties and proton conductivity compared to the pristine NbPBI membranes. The NbPBI-TSPDO membranes showed improved PA retention up to 81% at 160 ${}^{\circ }$C for 400 h in the phosphoric acid retention test.

Wang’s group reported [68] that three different ionic liquids ([VBIm]Cl, [PMIm]Br and [TPAm]Br) were crosslinked with NbPBI to prepare NbPBI-PIL membranes with three different crosslinking networks. The norbornene monomer of NbPBI could provide cross-linking sites, which combined with the ILs via in situ free radical reactions to cross-link. Moreover acid–base doping was the method to obtain dihydrogen phosphate type PIL cross-linked membranes. In comparison, NbPBI-MPIm membranes had better proton conductivity and phosphoric acid retention ability. Furthermore, the highest power density of NbPBI-TPAm membranes reached 385 mW·cm${}^{-2}$ in the cell test. All NbPBI-PIL membranes had high thermal stability and excellent mechanical properties, which showed that cross-linking modification by ionic liquids with polymer matrix was a promising research direction.

**Figure 5:**
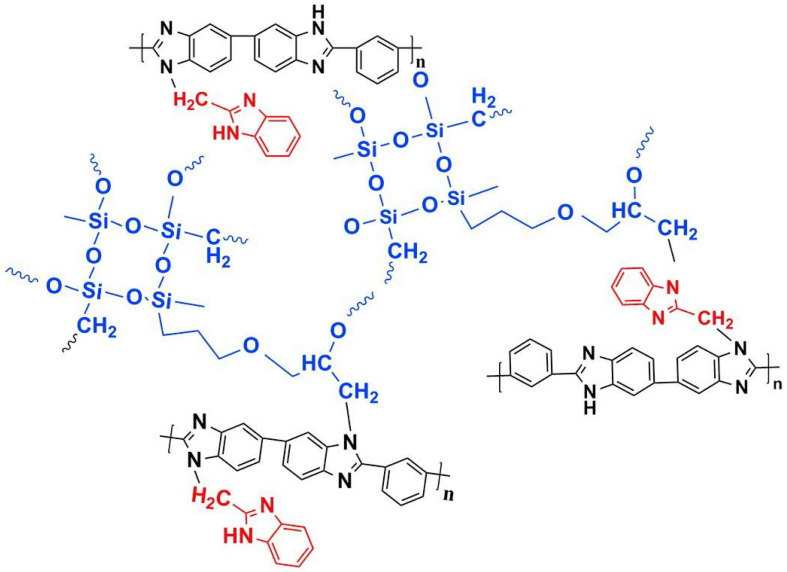
The cross-linked structure of CPBIm-X (taken from Ref. [69]).

Wang et al. reported that 2-chloromethyl benzimidazole (CMBelm) with imidazole groups [69] was first prepared and then grafted onto the PBI backbone to increase the imidazole group content of the substrate, while the side chain of the imidazole ring was flexible, which facilitated proton transport and reduced the activation energy. Then a series of CPBIm-X cross-linked membranes were prepared using 3-glycidoxypropyltrimethoxysilane (KH560) as a cross-linking agent (Figure 5). The imidazole groups of PBI could react with epoxy groups of KH560. KH560 could be hydrolyzed to form a Si-O-Si network structure, which could adsorb more phosphoric acid. Among them, the proton conductivity of CPBIm-5 was 0.092 S·cm${}^{-1}$, which was more than three times the proton conductivity of the original PBI membrane.

**Figure 6:**
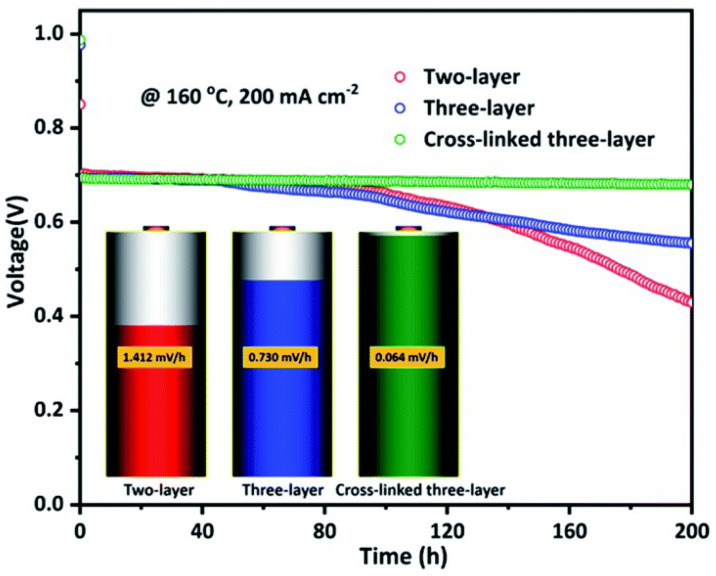
The voltage of different PBI-based membranes (taken from Ref. [70]).

It was reported by Wang’s group that a new leaf-like three-layer porous PA-doped PBI membrane [70] was designed and prepared. This membrane contained an internal porous layer and two dense skin layers as protective layers. This unique structure could improve the phosphoric acid adsorption level of the membrane. In addition, a cross-linked structure containing silicon was introduced into the porous PBI membrane, which greatly improved the durability property of the membrane. The PA-doped three-layer membrane had a high peak power density of 713 mW·cm${}^{-2}$ at 160 ${}^{\circ }$C. The PA-doped cross-linked three-layer membrane, on the other hand, exhibited good long-term stability resulting from silicon oxide formed from the hydrolysis of KH-560. The load voltage decay rate of this membrane was only 0.064 mV·h${}^{-1}$ (see Figure 6), which was about 8.8% of the decay of the three-layer membrane without the cross-linked structure.

**Figure 7:**
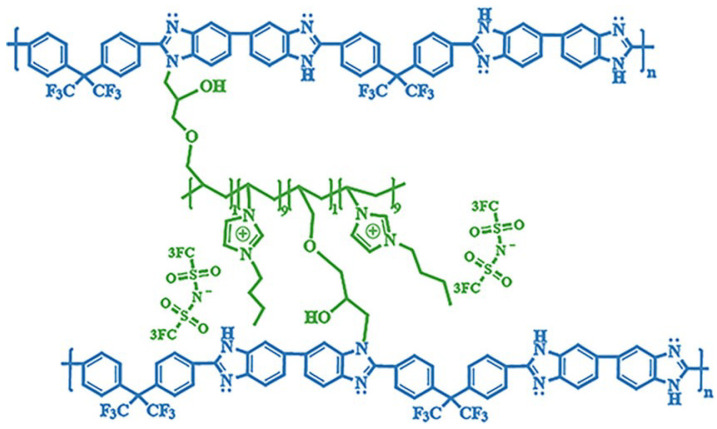
The cross-linked structure of 6FPBI-cPIL (taken from Ref. [71]).

In Wang’s work [71], the thermal cross-linking reaction of 6FPBI was carried out using cross-linkable polymeric ionic liquid (cPIL) as the cross-linking agent (Figure 7). PILs could form a continuous fast pathway for proton transmission via the anions on the PILs acting as proton acceptors. Meanwhile there was a synergistic effect between PILs and PBI that could result in enhanced physical and electrochemical properties. The most outstanding contribution of this work was that the 6FPBI-cPIL cross-linked membranes showed good phosphoric acid retention and long-term stability of proton conductivity stability even under extremely harsh conditions. In particular, the phosphoric acid adsorption level and proton conductivity of the 6FPBI-cPIL20 cross-linked membranes were consistently high at 8.5 and 0.064 S·cm${}^{-1}$, respectively. Later [75], based on the original work, a new ionic liquid PIL was replaced with 6FPBI as a substrate for the cross-linking reaction. The phosphoric acid adsorption level and the phosphoric acid retention were improved. There was 73.1% phosphoric acid retention ratio at 160 ${}^{\circ }$C/0 RH.

Lee et al. prepared new cross-linked ABPBI membranes via a direct casting process [72]. The cross-linked membranes can be changed by changing the ratio of reactants to change the degree of cross-linking of the cross-linked membranes. The effect of the chain entanglement after crosslinking could increase the mechanical stability of the membranes. The C-ABPBI-50 membrane was more stable than the ABPBI membrane which had an excellent chemical stability. The C-ABPBI-50 membrane had a proton conductivity of 0.12 S·cm${}^{-1}$ at 150 ${}^{\circ }$C.

Jana et al. [73] prepared cross-linked pyridine-bridged-oxypolybenzimidazole (PyOPBI) with bromomethyl polyphenylene oxide (BrPPO). The researchers synthesized a new kind of PyOPBI that had highly basic pyridine rings and large intermolecular distance between polymer chains, which had a bad stability in PA. They selected BrPPO as the cross-linker to form networks which reduced the free volume. A series of cross-linked membranes with different cross-linker contents were prepared by adjusting the cross-linker ratios. The cross-linked membranes were completely stable in 85% PA, while the uncross-linked PyOPBI membranes dissolved in 60% PA, showing the high stability of the cross-linked membranes. The P1 membrane exhibited a high proton conductivity of 0.123 S·cm${}^{-1}$ and a power density of 290 mW·cm${}^{-2}$. As a comparison, the original PyOPBI membrane had a proton conductivity of 0.008 S·cm${}^{-1}$ and a power density of 96.4 mW·cm${}^{-2}$.

Devrim et al. prepared four different PBI membranes which used four cross-linkers, bisphenol A diglycidyl ether (BADGE), ethylene glycol diglycidyl ether (EGDE), α-α${}^{’}$-dibromo-p-xylene (DBpX) and terephthalaldehyde (TPA) [63]. The researchers found that the cross-linked PBI membranes had better phosphoric acid retention properties compared to the PBI membrane. As a comparison with other membranes, the PBI/DBpX membrane had a high proton conductivity of 0.151 S·cm${}^{-1}$ at 180 ${}^{\circ }$C. The peak power density for PBI/BADGE was 123 mW·cm${}^{-2}$.

Wang et al. [74] established a covalently cross-linked PBI network using 2,6-Bis(hydroxy-methyl)-4-methylphenol (BHMP) as a cross-linking agent. The cross-linked network was established by N-substitution reaction between benzimidazole and hydroxy. Further, there was acidophilic hydroxyl group promoting the PA uptake on the cross-linked backbone. The covalently cross-linked network could improve the mechanical properties, proton conduction and fuel cell property of the cross-linked membrane. The covalently cross-linked membrane with 1% BHMP exhibited a high proton conductivity of 168.4 S·cm${}^{-1}$. It also exhibited a good power density of 597.5 mW·cm${}^{-2}$ at 160 ${}^{\circ }$C without humidification. On the other hand, the voltage decay of the cross-linked membrane at a current density of 200 mA·cm${}^{-2}$ was also low at 0.0212 mV·h${}^{-1}$. This indicated that the cross-linked membrane had good stability.

**Figure 8:**
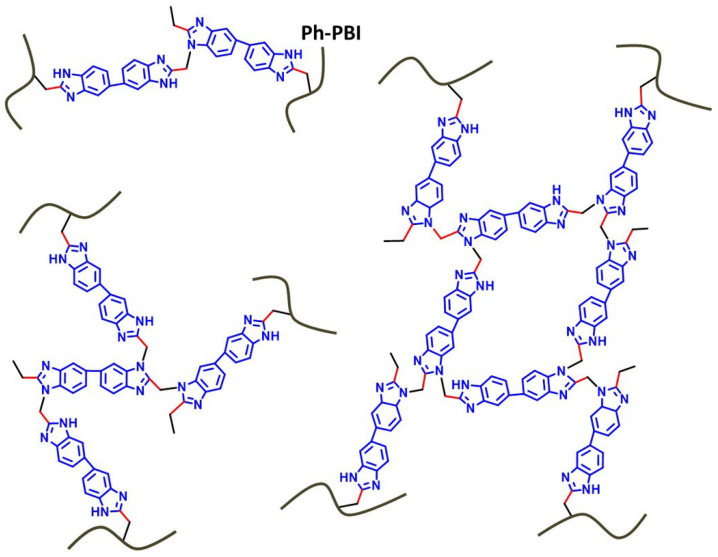
The cross-linked structure of 2BIM-2Cl in Ph-PBI (taken from Ref. [65]).

In Liu’s work [65], the cross-linking agent 2BIM-2Cl was incorporated into Ph-PBI to construct a cross-linked network rich in imidazole groups, making the system highly resistant to plasticization (Figure 8). Unusually, its cross-linking agent A_2_B_2_ could not only have the effect of cross-linking polymers, but also had the dramatic effect of self-reaction. Generally speaking, the content of the imidazole groups would be greatly reduced after cross-linking but the 2BIM-2Cl had many imidazole groups which could enhance PA adsorption. The cross-linker could be added to the membrane in large quantities without degrading the properties of the membrane. Cross-linked membranes exhibited high ADL value, proton conductivity and stability due to the high content of imidazole groups. One of the cross-linked membranes with 30% cross-linker had 100 wt% enhanced phosphoric acid adsorption level and doubled proton conductivity at 200 ${}^{\circ }$C. The highest cell power density was 533 mW·cm${}^{-2}$.

It is not difficult to find that the mechanical strength and dimensional stability of the reported cross-linked PBI proton exchange membranes have been improved to a certain extent, but the introduction of cross-linked components inevitably reduces the relative content of functional imidazole groups, and the cross-linking leads to tightly packed PBI molecular chains, which makes them generally face the insurmountable problem of reduced acid absorption or proton conductivity. The construction of an ideal PBI cross-linking network requires the design of structurally sound and mutually compatible substrate and cross-linker components, as well as an efficient and controlled cross-linking reaction between them. In the structure of the PBI molecule, it is the imidazole group that plays a decisive role in proton conduction. In general, the introduction of cross-linking agents leads to a decrease in the content of imidazole groups, which affects the proton conduction. As can be seen from the above examples, the cross-linking agent 2BIM-2Cl has been very successful. It is inherently rich in imidazole groups and does not decrease the content of imidazole groups due to the introduction of cross-linking agents. At the same time, it also has a self-cross-linking effect and does not cause degradation of performance due to excessive addition of cross-linking agent.

## 4. Cross-Linked Polybenzimidazole-Based Composite and Blend Membranes

After studying the cross-linking reaction between PBI and cross-linkers to enhance the properties of membranes, researchers have tried to dope the cross-linked PBI with some substances to prepare a series of distinctive cross-linked PBI-based composite or blend membranes. It is obvious that the prepared cross-linked PBI composite and blend membranes may have some improved properties by both cross-linking and doping. This chapter shows properties of cross-linked polybenzimidazole composite and blend membranes, as summarized in Table 3.

A series of blend membranes cross-linked with poly (vinylbenzyl chloride) and OPBI and selected with three amines (DABCO, quinuclidine and quinuclidinol) introduced into the system were reported to be prepared in Bohm’s work [76]. The cross-linked membranes had good dimensional stability and better mechanical properties. By adjusting the ratio of OPBI and PVBC and then adding three different amines separately to perform various tests, the best performing membrane reached 530 mW·cm${}^{-2}$ at 180 ${}^{\circ }$C. This was much better than the original OPBI membrane.

**Figure 9:**
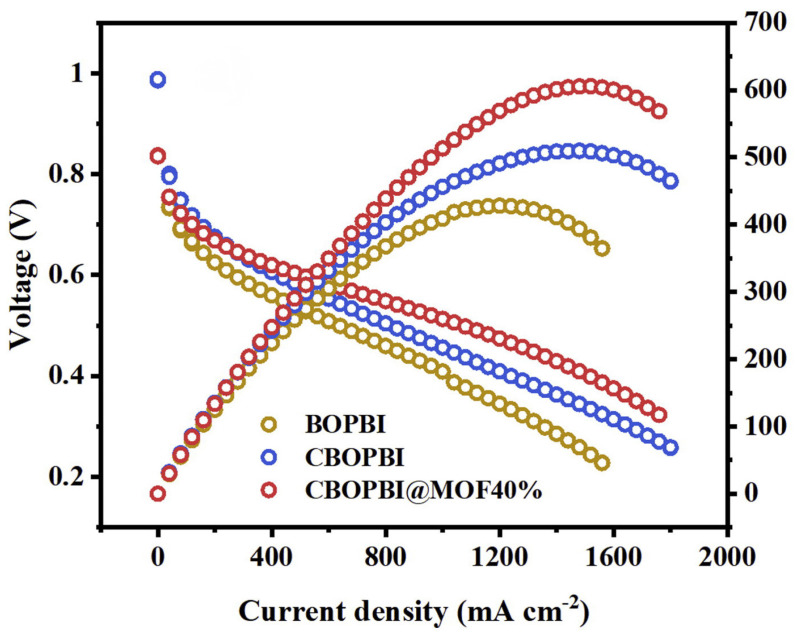
The voltage of CBOPBI@MOF40% (taken from Ref. [77]).

Wang at al. pointed out that the cross-linked branched PBI was chosen as the polymer matrix considering that the cross-linked structure had good chemical stability and high proton conductivity [77]. Then UiO-66 (MOF) was introduced into the structure to construct the proton transport channels. A series of CBOPBI composite membranes with different mass percentages of UiO-66 were successfully prepared. Among them, the CBOPBI@MOF 40% membrane showed the lowest absorption of 126% for PA and reached the highest proton conductivity of 0.100 S·cm${}^{-1}$ at 160 ${}^{\circ }$C, which possessed an excellent peak power density of 607 mW·cm${}^{-2}$ (Figure 9).

A multifunctional cross-linkable proton conductor (TTPSA) was reported [78] to prepare a series of cross-linked proton exchange membranes by multiple cross-linking of chloromethylated polyetherimide (CMPEI) and mPBI in Hui’s work. The three components had good miscibility, which could reduce phase separation. As the proton conductor, TTPSA in particular could be the cross-linker in the cross-linked network. Among them, CMPEI acted as a bridge to connect mPBI and TTPSA. There were three kinds of cross-linking methods, covalent, ionic and hydrogen bonds in the cross-link network, resulting in less leaching of proton conductor. There were many basic nitrogen sites in the cross-linked network to enhance the hydrogen bonding interactions, thus improving the stability property of the blend membranes. mPBI-CMPEI(20)-TTPSA(30) achieved a proton conductivity of 0.113 S·cm${}^{-1}$ at 180 ${}^{\circ }$C and 100% RH. More importantly, even after 48 h of washing, the proton conductivity remained almost unchanged, decreasing by only 0.4%.

The cross-linking agents from the two aforementioned studies were used in combination in Wang’s studies [79]. First, a cross-linked composite membrane of PBI and PIL was prepared, and then KH560 was introduced into the matrix to enhance the membrane. The concept of a dual proton transfer channel was proposed, in which the anion of PIL acted as a proton acceptor through electrostatic interaction and then the imidazole ring accepted the proton through acid–base interaction. Then the mechanical properties of the membrane were enhanced by the hydrolysis of KH560, which generated the Si-O-Si network structure. Among them, cPBI-BF_4_-40 exhibited a proton conductivity of 0.117 S·cm${}^{-1}$ at 170 ${}^{\circ }$C, which reached a balance between mechanical strength and phosphoric acid adsorption.

It was reported that 6-tert-butyl-3-phenyl-3,4-dihydro-2H-benzo[e] [1,3] oxazine (*p*BUa) was cross-linked with PBI via covalent cross-linking [80]. The covalently cross-linked structure resulted from the carbon of the benzene ring in the P(*p*BUa) being bonded to the benzene ring in PBI. The chemical stability of blend membranes was better than the PBI membranes. The proton conductivity of the P(*p*BUa-*co*-BI)-65 membrane was 0.121 S·cm${}^{-1}$ at 150 ${}^{\circ }$C under un-humidified conditions. The peak power density of the P(*p*BUa-*co*-BI)-65 membrane nearly reached 430 mW·cm${}^{-2}$.

In Yin’s work [81], TGDDM was first covalently cross-linked with mPBI and then doped with ZrSP in the blend membranes. TGDDM had multi-group crosslinking groups, which could reduce the use of cross-linker. The cross-link network was a hydrogen-bonding network formed by mPBI (amido group), TGDDM (epoxy group) and ZrSP (sulfonic group). The system contained a large amount of sulfonic acid group and phosphoric acid group. The dimensional stability, phosphoric acid adsorption level and proton conductivity of the membrane were improved at low cross-linking. In particular, the proton conductivity of mPBI-TGDDM (5%)/ZrSP (50%) membranes was 0.127 S·cm${}^{-1}$ at 180 ${}^{\circ }$C at 100% relative humidity (RH).

In another work by Yin [82], a series of cross-linked membranes with different contents were prepared by cross-linking PBI with TGIC after doping with SPAN. TGIC was a trifunctional cross-linker with a relatively low cross-linking degree, thus enabling SPAN to be uniformly dispersed in the matrix. The hydrogen bonding networks were established by PBI (imidazole rings), TGIC (amide groups and hydroxyl groups) and SPAN (sulfonic groups and amine groups). The linear PBI formed a three-dimensional network structure, which greatly improved the mechanical properties, dimensional stability and proton conductivity. PBI-TGIC(5%)/SPAN had a tensile strength of 21–41.5 MPa. The cross-linked blend membranes also exhibited very low methanol permeability and membrane selectivity. Similarly, in another work [84], a series of cross-linked blend membranes were prepared by cross-linking PBI with TGIC and then doping with SPOP. The good compatibility of SPOP with mPBI-TGIC led to its uniform dispersion in the membranes without phase separation structure. The proton conductivity of the membranes was improved. Among them, the conductivity of mPBI-TGIC (5%)/SPOP (50%) was 0.143 S·cm${}^{-1}$ at 180 ${}^{\circ }$C at 100% RH.

**Figure 10:**
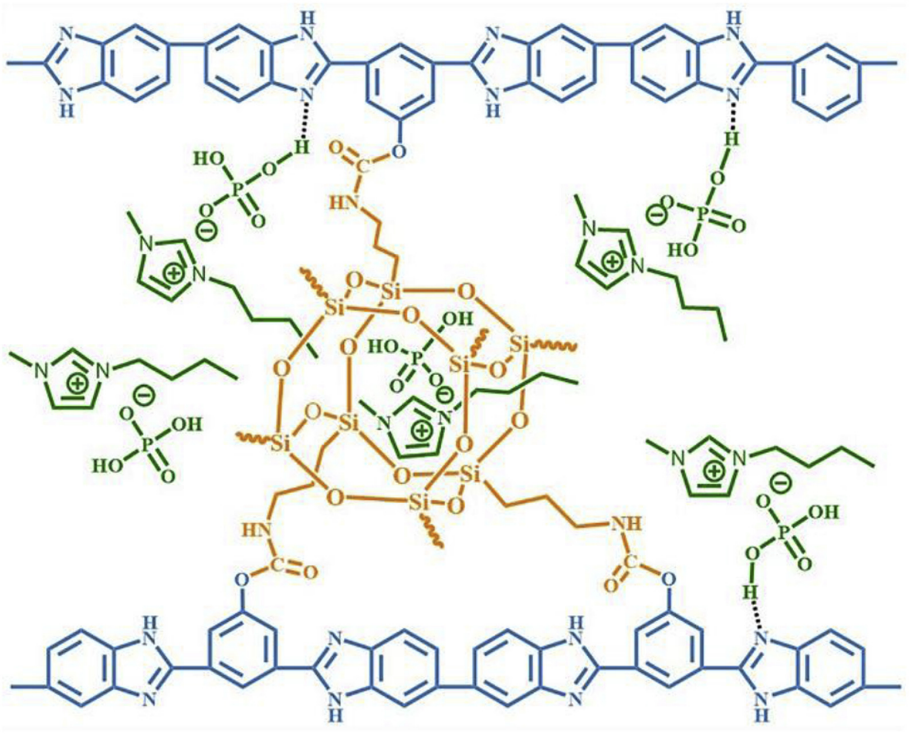
The cross-linked structure of cPBI-IL X (taken from Ref. [83]).

Wang’s group reported that a series of polybenzimidazole/ionic liquid composite membranes with cage-like cross-linked structures (Figure 10) were prepared from hydroxyl-containing polybenzimidazole, 3-(triethoxysilyl) propyl isocyanate and 1-butyl-3-methylimidazolium dihydrogen phosphate (BMI-DHPH) [83]. The imidazole ring in the ionic liquid greatly enhanced the phosphoric acid adsorption level. The cage-like cross-linked structure could be formed under acidic conditions, which could improve the retention ability of ionic liquids. After 144 h of IL retention test, the residual rate of IL in cPBI-IL 10 was 72.6%, which showed its strong retention ability. cPBI-IL 8 had a proton conductivity of 0.133 S·cm${}^{-1}$ at 160 ${}^{\circ }$C.

**Figure 11:**
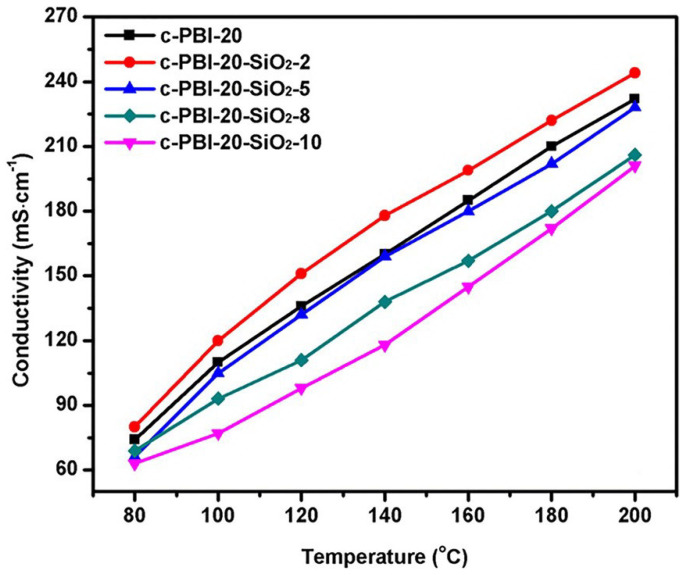
The proton conductivity of the SiO_2_/c-PBI-20 composite membranes (taken from Ref. [85]).

Liu’s group [85] also dispersed porous polyhydroxy SiO_2_ nanoparticles into the cross-linked network, which greatly enhanced the phosphoric acid retention ability. The proton conductivity of c-PBI-20-SiO_2_-2 composite membranes is 0.244 S·cm${}^{-1}$ (Figure 11). Their cross-linked membranes showed a cell power density of 497 mW·cm${}^{-2}$ at 160 ${}^{\circ }$C under anhydrous conditions.

In contrast to the simple cross-linked system construction in the previous chapter, other modifications are made to the cross-linked system in this chapter. By introducing hyperbranched structures or nanoscale “pore” microstructures into the cross-linked membranes, the cross-linked systems with micro-phase adjustment capability are constructed. The new system can solve many problems of current high-temperature proton exchange membranes, and the new cross-linked membranes have low swelling and high resistance to plasticization at high phosphoric acid doping levels. The research ideas in this chapter provide valuable information for the design and preparation of future high-performance HT-PEMFCs.

## 5. Conclusions

Cross-linking is an essential method for modification of polybenzimidazole-type membranes. The linear polybenzimidazole polymers become a cross-linked polybenzimidazole-based network, which results in a significant enhancement of some physico-chemical properties. Generally, cross-linked polybenzimidazole-based membranes may have improved stability, mechanical properties, phosphoric acid adsorption and retention levels, proton conductivity, and cell properties compared to pristine polybenzimidazole membranes. A variety of cross-linking systems are available and the key is to select a cross-linker rich in functional groups that facilitate proton conduction. On the other hand, a microenvironment favorable for proton conduction and phosphoric acid adsorption and retention is established on the basis of cross-linking. It is necessary to carry out an exploratory study of phosphoric acid storage and retention methods with microscopic phase regulation capability. For example, the introduction of substances containing pores into the cross-linked membrane systems can greatly enhance the phosphoric acid adsorption and retention levels, ultimately improving the proton conductivity and cell properties of the cross-linked membrane. Therefore, the fabrication of cross-linked polybenzimidazole-based membranes is an important direction for future research in the field of high-temperature proton exchange membranes.

As people pay more attention to the energy crisis and environmental protection, the exploration and development of environmentally friendly new energy sources such as fuel cells will continue. This review summarizes new cross-linked PBI high-temperature proton exchange membranes with good properties and potential for fuel cell applications. As the most promising high-temperature proton exchange membrane, more and more new materials and applications have emerged, theories and experiments are developing rapidly, and research ideas are broadening. It can be believed that polybenzimidazole will definitely achieve large-scale applications in the future. However, fuel cells are actually system engineering, and further exploration is needed for testing the overall fuel cell properties and practical applications in fuel cells.

## Figures and Tables

**Table 1:** Some examples of the polymer membranes for fuel cells.

Membrane	Fuel Cell Type	Test Temperature (${}^{\circ }$C)	Proton Conductivity (S·cm${}^{-1}$)	Peak Power Density (mW·cm${}^{-2}$)	Chemical Structure
PES-P5-1.2 [20]	AEMFC	20–80	0.053 (80 ${}^{\circ }$C)	135 (60 ${}^{\circ }$C)	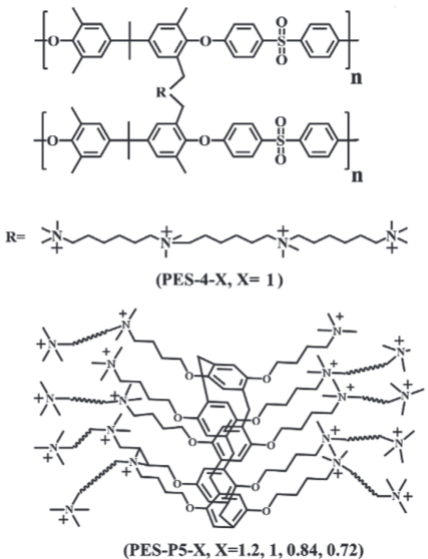
PES-P5-12 [21]	AEMFC	20–80	0.093 (80 ${}^{\circ }$C)	105 (60 ${}^{\circ }$C)	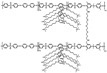
PPO-30-15CD [22]	AEMFC	20–80	0.099 (80 ${}^{\circ }$C)	154 (60 ${}^{\circ }$C)	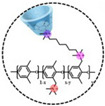
Orion TM1 [23]	AEMFC	20–80	0.125 (50 ${}^{\circ }$C)	-	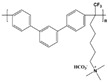
c-PES-12.8-PPO-20 [24]	AEMFC	20–80	0.126 (80 ${}^{\circ }$C)	-	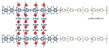
PPO-HVBC-100 [25]	AEMFC	25–80	0.136 (80 ${}^{\circ }$C)	71 (60 ${}^{\circ }$C)	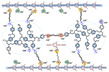
PAM/SPAEK/POSS [26]	LT-PEMFC	20–80	0.031 (80 ${}^{\circ }$C)	-	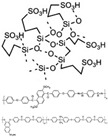
CF_3_-SPI-0 [27]	LT-PEMFC	20–100	0.085 (80 ${}^{\circ }$C)	-	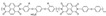
SP-SPI/POSS [28]	LT-PEMFC	20–100	0.144 (100 ${}^{\circ }$C)	-	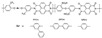
SC-SPAEK/TiO_2_-4 [29]	LT-PEMFC	20–100	0.147 (100 ${}^{\circ }$C)	-	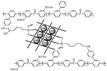
CN-SPI-4 [30]	LT-PEMFC	20–100	0.149 (100 ${}^{\circ }$C)	-	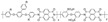
Nafion 117 [30]	LT-PEMFC	20–100	0.156 (100 ${}^{\circ }$C)	-	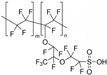
PBI/SPAEK-SPOSS-1% [31]	HT-PEMFC	100–200	0.126 (200 ${}^{\circ }$C)	300 (160 ${}^{\circ }$C)	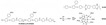
OPBI/H-VBC-QA-1 [32]	HT-PEMFC	100–200	0.152 (200 ${}^{\circ }$C)	-	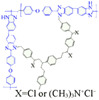
PBI/Allyl-SPAEK-10% [33]	HT-PEMFC	100–200	0.206 (200 ${}^{\circ }$C)	-	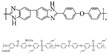
g-PBI-20 [34]	HT-PEMFC	100–200	0.212 (200 ${}^{\circ }$C)	443 (160 ${}^{\circ }$C)	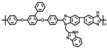
OPBI [35]	HT-PEMFC	100–200	0.25 (200 ${}^{\circ }$C)	161 (160 ${}^{\circ }$C)	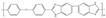
OPBI/PIM-1(10%) [35]	HT-PEMFC	100–200	0.313 (200 ${}^{\circ }$C)	438 (160 ${}^{\circ }$C)	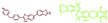

**Table 2:** Some cross-linked polybenzimidazole membranes.

Membrane	Cross-Linking Method	Proton Conductivity (S·cm${}^{-1}$)	Peak Power Density (mW·cm${}^{-2}$)	Chemical Structure of PBI and Cross-Linker
QOPBI-15 [66]	N-substitution reactions	0.049 (160 ${}^{\circ }$C)	260 (160 ${}^{\circ }$C)	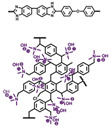
NbPBI-TSPDO_30_ [67]	Acid-base interactions	0.061 (170 ${}^{\circ }$C)	159 (120 ${}^{\circ }$C)	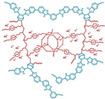
NbPBI-MPIm [68]	Acid-base interactions	0.074 (170 ${}^{\circ }$C)	375 (160 ${}^{\circ }$C)	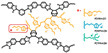
CPBIm-5 [69]	N-substitution reactions	0.092 (180 ${}^{\circ }$C)	-	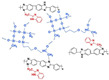
Cross-linked three-layer [70]	N-substitution reactions	0.101 (200 ${}^{\circ }$C)	605 (160 ${}^{\circ }$C)	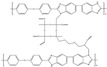
6FPBI-cPIL20 [71]	N-substitution reactions	0.106 (170 ${}^{\circ }$C)	-	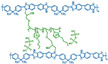
C-ABPBI-50 [72]	Covalent cross-linking	0.12 (150 ${}^{\circ }$C)	-	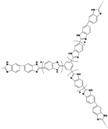
P1 [73]	N-substitution reactions	0.123 (180 ${}^{\circ }$C)	289 (160 ${}^{\circ }$C)	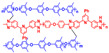
PBI/DBpX [63]	N-substitution reactions	0.151 (180 ${}^{\circ }$C)	106 (165 ${}^{\circ }$C)	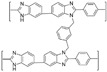
1%-OPBI [74]	N-substitution reactions	0.168 (160 ${}^{\circ }$C)	598 (160 ${}^{\circ }$C)	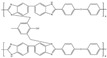
c-PBI-30 [65]	N-substitution reactions	0.253 (200 ${}^{\circ }$C)	533 (160 ${}^{\circ }$C)	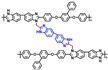

**Table 3:** Some cross-linked polybenzimidazole-based composite and blend membranes.

Membrane	Cross-Linking Method	Proton Conductivity (S·cm${}^{-1}$)	Peak Power Density (mW·cm${}^{-2}$)	Chemical Structure of PBI and Cross-Linker
60-40-Q [76]	Covalent and ionic cross-linking	0.092 (180 ${}^{\circ }$C)	530 (180 ${}^{\circ }$C)	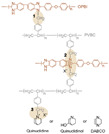
CBOPBI@MOF 40% [77]	N-substitution reactions	0.1 (160 ${}^{\circ }$C)	607 (160 ${}^{\circ }$C)	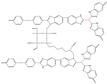
mPBI-CMPEI(20)-TTPSA(30) [78]	N-substitution reactions and covalent cross-linking	0.113 (180 ${}^{\circ }$C)	-	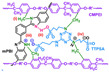
cPBI-BF_4_-40 [79]	N-substitution reactions	0.117 (170 ${}^{\circ }$C)	-	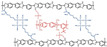
P(*p*BUa-*co*-BI)-65 [80]	Covalent cross-linking	0.121 (150 ${}^{\circ }$C)	410 (150 ${}^{\circ }$C)	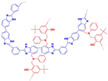
mPBI-TGDDM (5%)/ZrSP (50%) [81]	N-substitution reactions and covalent cross-linking	0.127 (180 ${}^{\circ }$C)	-	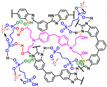
PBI-TGIC(5%)/SPAN(50%) [82]	N-substitution reactions	0.13 (180 ${}^{\circ }$C)	-	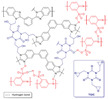
cPBI-IL 8 [83]	Covalent cross-linking	0.133 (160 ${}^{\circ }$C)	-	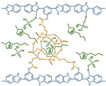
mPBI-TGIC (5%)/SPOP (50%) [84]	N-substitution reactions	0.143 (180 ${}^{\circ }$C)	-	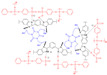
c-PBI-20-SiO_2_-2 [85]	N-substitution reactions	0.244 (200 ${}^{\circ }$C)	497 (160 ${}^{\circ }$C)	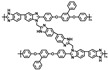

## Data Availability

Not applicable. No new data were created or analyzed in this study. Data sharing is not applicable to this article.

## References

[1] Krishnan N.N., Konovalova A., Aili D., Li Q., Park H.S., Jang J.H., Kim H.-J., Henkensmeier D. (2019). Thermally crosslinked sulfonated polybenzimidazole membranes and their performance in high temperature polymer electrolyte fuel cells. J. Membr. Sci..

[2] Jiao K., Xuan J., Du Q., Bao Z., Xie B., Wang B., Zhao Y., Fan L., Wang H., Hou Z. (2021). Designing the next generation of proton-exchange membrane fuel cells. Nature.

[3] Araya S.S., Zhou F., Liso V., Sahlin S.L., Vang J.R., Thomas S., Gao X., Jeppesen C., Kaer S.K. (2016). A comprehensive review of PBI-based high temperature PEM fuel cells. Int. J. Hydrogen Energy.

[4] Kalathil A., Raghavan A., Kandasubramanian B. (2019). Polymer Fuel Cell Based on Polybenzimidazole Membrane: A Review. Polym-Plast. Tech. Mat..

[5] Maiti T.K., Singh J., Majhi J., Ahuja A., Maiti S., Dixit P., Bhushan S., Bandyopadhyay A., Chattopadhyay S. (2022). Advances in polybenzimidazole based membranes for fuel cell applications that overcome Nafion membranes constraints. Polymer.

[6] Wang P., Li X., Liu Z., Peng J., Shi C., Li T., Yang J., Shan C., Hu W., Liu B. (2022). Construction of highly conductive PBI-based alloy membranes by incorporating PIMs with optimized molecular weights for high-temperature proton exchange membrane fuel cells. J. Membr. Sci..

[7] Yu S., Xiao L., Benicewicz B.C. (2008). Durability studies of PBI-based high temperature PEMFCs. Fuel Cells.

[8] Altaf F., Batool R., Gill R., Rehman Z.U., Majeed H., Ahmad A., Shafiq M., Dastan D., Abbas G., Jacob K. (2021). Synthesis and electrochemical investigations of ABPBI grafted montmorillonite based polymer electrolyte membranes for PEMFC applications. Renew. Energ..

[9] Ma Y.L., Wainright J.S., Litt M.H., Savinell R.F. (2004). Conductivity of PBI membranes for high-temperature polymer electrolyte fuel cells. J. Electrochem. Soc..

[10] Cheddie D., Munroe N. (2006). Mathematical model of a PEMFC using a PBI membrane. Energy Convers. Manag..

[11] Park J.O., Kwon K., Cho M.D., Hong S.G., Kim T.Y., Yoo D.Y. (2011). Role of Binders in High Temperature PEMFC Electrode. J. Electrochem. Soc..

[12] Sun X., Simonsen S.C., Norby T., Chatzitakis A. (2019). Composite Membranes for High Temperature PEM Fuel Cells and Electrolysers: A Critical Review. Membranes.

[13] Quartarone E., Angioni S., Mustarelli P. (2017). Polymer and Composite Membranes for Proton-Conducting, High-Temperature Fuel Cells: A Critical Review. Materials.

[14] Yusoff Y.N., Loh K.S., Wong W.Y., Daud W.R.W., Lee T.K. (2020). Sulfonated graphene oxide as an inorganic filler in promoting the properties of a polybenzimidazole membrane as a high temperature proton exchange membrane. Int. J. Hydrogen Energy.

[15] Venugopalan G., Chang K., Nijoka J., Livingston S., Geise G.M., Arges C.G. (2020). Stable and Highly Conductive Polycation-Polybenzimidazole Membrane Blends for Intermediate Temperature Polymer Electrolyte Membrane Fuel Cells. ACS. Appl. Energ. Mater..

[16] Escorihuela J., Olvera-Mancilla J., Alexandrova L., del Castillo L.F., Compan V. (2020). Recent Progress in the Development of Composite Membranes Based on Polybenzimidazole for High Temperature Proton Exchange Membrane (PEM) Fuel Cell Applications. Polymers.

[17] Seland F., Berning T., Borresen B., Tunold R. (2006). Improving the performance of high-temperature PEM fuel cells based on PBI electrolyte. J. Power Sources.

[18] Bose S., Kuila T., Thi Xuan Lien N., Kim N.H., Lau K.-T., Lee J.H. (2011). Polymer membranes for high temperature proton exchange membrane fuel cell: Recent advances and challenges. Prog. Polym. Sci..

[19] Lee J., Jung J., Han J.Y., Kim H.-J., Jang J.H., Lee H.-J., Cho E.A., Henkensmeier D., Kim J.Y., Yoo S.J. (2014). Effect of Membrane Electrode Assembly Fabrication Method on the Single Cell Performances of Polybenzimidazole-Based High Temperature Polymer Electrolyte Membrane Fuel Cells. Macromol. Res..

[20] Peng J., Liang M., Cao K., Liu Z., Wang P., Hu W., Jiang Z., Liu B. (2020). Fabrication of Cross-Linked Anion Exchange Membranes Using a Pillar 5 arene Bearing Multiple Alkyl Bromide Head Groups as Cross-Linker. Macromol. Mater. Eng..

[21] Peng J., Liang M., Liu Z., Wang P., Shi C., Hu W., Liu B. (2020). Poly(arylene ether sulfone) crosslinked networks with pillar 5 arene units grafted by multiple long-chain quaternary ammonium salts for anion exchange membranes. Chem. Commun..

[22] Liang M., Peng J., Cao K., Shan C., Liu Z., Wang P., Hu W., Liu B. (2022). Multiply quaternized poly(phenylene oxide)s bearing beta-cyclodextrin pendants as “assisting moiety” for high-performance anion exchange membranes. J. Membr. Sci..

[23] Kim S., Kwon H., Lee H., Jung N., Bae B., Shin D. (2022). Electrochemical Method for Measurement of Hydroxide Ion Conductivity and CO_2_ Poisoning Behavior of Anion Exchange Membrane. J. Korean Electrochem. Soc..

[24] Peng J., Liu Z., Liang M., Wang P., Hu W., Jiang Z., Liu B. (2020). Highly conductive and stable anion-exchange membranes based on crosslinked poly(arylene ether sulfone)-block-poly(phenylene oxide) Networks. J. Polym. Sci..

[25] Cao K., Peng J., Shan C., Liu Z., Liang M., Wang L., Hu W., Liu B. (2022). Beneficial use of hyperbranched polymer in cross-linked anion exchange membranes for fuel cells. Int. J. Energy Res..

[26] Zhu M., Song Y., Hu W., Li X., Jiang Z., Guiver M.D., Liu B. (2012). SPAEK-based binary blends and ternary composites as proton exchange membranes for DMFCs. J. Membr. Sci..

[27] Song Y., Liu C., Ren D., Jing L., Jiang Z., Liu B. (2013). Fluorinated/non-fluorinated sulfonated polynaphthalimides as proton exchange membranes. Macromol. Res..

[28] Song Y., Cao X., Liang Q., Jin Y., Qi Y., Hu W., Li K., Jiang Z., Liu B. (2014). Sulfonated polyimides and their polysilsesquioxane hybrid membranes for fuel cells. Solid State Ionics.

[29] Liu Z., Wang P., Hu W., Liu B. (2019). Preparation and Properties of Hybrid Silane-crosslinked Sulfonated Poly(aryl ether ketone)s as Proton Exchange Membranes. Chem. Res. Chin. Univ..

[30] Song Y., Jin Y., Liang Q., Li K., Zhang Y., Hu W., Jiang Z., Liu B. (2013). Novel sulfonated polyimides containing multiple cyano groups for polymer electrolyte membranes. J. Power Sources.

[31] Yang J., Li X., Shi C., Liu B., Cao K., Shan C., Hu W., Liu B. (2021). Fabrication of PBI/SPOSS hybrid high-temperature proton exchange membranes using SPAEK as compatibilizer. J. Membr. Sci..

[32] Cao K., Peng J., Li H., Shi C., Wang P., Liu B. (2021). High-temperature Proton Exchange Membranes Based on Cross-linked Polybenzimidazole/hyperbranched-polymer Blends. Chem. J. Chin. Univ..

[33] Liang M., Wang P., Li H., Li T., Cao K., Peng J., Liu Z., Liu B. (2020). Preparation of High-temperature Proton Exchange Membranes Based on Semi-interpenetrating Polymer Networks. Chem. J. Chin. Univ..

[34] Li X., Wang P., Liu Z., Peng J., Shi C., Hu W., Jiang Z., Liu B. (2018). Arylether-type polybenzimidazoles bearing benzimidazolyl pendants for high-temperature proton exchange membrane fuel cells. J. Power Sources.

[35] Wang P., Liu Z., Li X., Peng J., Hu W., Liu B. (2019). Toward enhanced conductivity of high-temperature proton exchange membranes: Development of novel PIM-1 reinforced PBI alloy membranes. Chem. Commun..

[36] Staiti P., Minutoli M., Hocevar S. (2000). Membranes based on phosphotungstic acid and polybenzimidazole for fuel cell application. J. Power Sources.

[37] Matar S., Higier A., Liu H. (2010). The effects of excess phosphoric acid in a Polybenzimidazole-based high temperature proton exchange membrane fuel cell. J. Power Sources.

[38] Jahangiri S., Aravi I., Sanli L.I., Menceloglu Y.Z., Ozden-Yenigun E. (2018). Fabrication and optimization of proton conductive polybenzimidazole electrospun nanofiber membranes. Polym. Adv. Technol..

[39] van de Ven E., Chairuna A., Merle G., Benito S.P., Borneman Z., Nijmeijer K. (2013). Ionic liquid doped polybenzimidazole membranes for high temperature Proton Exchange Membrane fuel cell applications. J. Power Sources.

[40] Jang J., Kim D.-H., Ahn M.-K., Min C.-M., Lee S.-B., Byun J., Pak C., Lee J.-S. (2020). Phosphoric acid doped triazole-containing cross-linked polymer electrolytes with enhanced stability for high-temperature proton exchange membrane fuel cells. J. Membr. Sci..

[41] Wainright J.S., Wang J.T., Weng D., Savinell R.F., Litt M. (1995). Acid-Doped Polybenzimidazoles: A New Polymer Electrolyte. J. Electrochem. Soc..

[42] Li Q., Jensen J.O., Savinell R.F., Bjerrum N.J. (2009). High temperature proton exchange membranes based on polybenzimidazoles for fuel cells. Prog. Polym. Sci..

[43] Chen J., Wang L., Wang L. (2020). Highly Conductive Polybenzimidazole Membranes at Low Phosphoric Acid Uptake with Excellent Fuel Cell Performances by Constructing Long-Range Continuous Proton Transport Channels Using a Metal-Organic Framework (UIO-66). ACS. Appl. Mater. Inter..

[44] Escorihuela J., Garcia-Bernabe A., Montero A., Sahuquillo O., Gimenez E., Compan V. (2019). Ionic Liquid Composite Polybenzimidazol Membranes for High Temperature PEMFC Applications. Polymers.

[45] Hooshyari K., Javanbakht M., Adibi M. (2016). Novel composite membranes based on dicationic ionic liquid and polybenzimidazole mixtures as strategy for enhancing thermal and electrochemical properties of proton exchange membrane fuel cells applications at high temperature. Int. J. Hydrogen Energy.

[46] Haider R., Wen Y., Ma Z.-F., Wilkinson D.P., Zhang L., Yuan X., Song S., Zhang J. (2021). High temperature proton exchange membrane fuel cells: Progress in advanced materials and key technologies. Chem. Soc. Rev..

[47] Wannek C., Konradi I., Mergel J., Lehnert W. (2009). Redistribution of phosphoric acid in membrane electrode assemblies for high-temperature polymer electrolyte fuel cells. Int. J. Hydrogen Energy.

[48] Lobato J., Canizares P., Rodrigo M.A., Ubeda D., Javier Pinar F. (2011). A novel titanium PBI-based composite membrane for high temperature PEMFCs. J. Membr. Sci..

[49] Hooshyari K., Rezania H., Vatanpour V., Salarizadeh P., Askari M.B., Beydaghi H., Enhessari M. (2020). High temperature membranes based on PBI/sulfonated polyimide and doped-perovskite nanoparticles for PEM fuel cells. J. Membr. Sci..

[50] Lee S., Seo K., Ghorpade R.V., Nam K.-H., Han H. (2020). High temperature anhydrous proton exchange membranes based on chemically-functionalized titanium/polybenzimidazole composites for fuel cells. Mater. Lett..

[51] He R., Che Q., Sun B. (2008). The acid doping behavior of polybenzimidazole membranes in phosphoric acid for proton exchange membrane fuel cells. Fiber. Polym..

[52] Tang H., Geng K., Wu L., Liu J., Chen Z., You W., Yan F., Guiver M.D., Li N. (2022). Fuel cells with an operational range of −20 °C to 200 °C enabled by phosphoric acid-doped intrinsically ultramicroporous membranes. Nat. Energy.

[53] Wang D., Wang S., Tian X., Li J., Liu F., Wang X., Chen H., Mao T., Liu G. (2020). Ethyl phosphoric acid grafted amino-modified polybenzimidazole with improved long-term stability for high-temperature proton exchange membrane applications. Int. J. Hydrogen Energy.

[54] Rath R., Kumar P., Unnikrishnan L., Mohanty S., Nayak S.K. (2020). Current Scenario of Poly (2,5-Benzimidazole) (ABPBI) as Prospective PEM for Application in HT-PEMFC. Polym. Rev..

[55] Li Q.F., He R.H., Jensen J.O., Bjerrum N.J. (2003). Approaches and recent development of polymer electrolyte membranes for fuel cells operating above 100 °C. Chem. Mater..

[56] Berber M.R., Nakashima N. (2019). Bipyridine-based polybenzimidazole membranes with outstanding hydrogen fuel cell performance at high temperature and non-humidifying conditions. J. Membr. Sci..

[57] Mader J.A., Benicewicz B.C. (2010). Sulfonated Polybenzimidazoles for High Temperature PEM Fuel Cells. Macromolecules.

[58] Li X., Ma H., Shen Y., Hu W., Jiang Z., Liu B., Guiver M.D. (2016). Dimensionally-stable phosphoric acid-doped polybenzimidazoles for high-temperature proton exchange membrane fuel cells. J. Power Sources.

[59] Ni J., Hu M., Liu D., Xie H., Xiang X., Wang L. (2016). Synthesis and properties of highly branched polybenzimidazoles as proton exchange membranes for high-temperature fuel cells. J. Mater. Chem. C.

[60] Li Q., Pan C., Jensen J.O., Noye P., Bjerrum N.J. (2007). Cross-linked polybenzimidazole membranes for fuel cells. Chem. Mater..

[61] Han M., Zhang G., Liu Z., Wang S., Li M., Zhu J., Li H., Zhang Y., Lew C.M., Na H. (2011). Cross-linked polybenzimidazole with enhanced stability for high temperature proton exchange membrane fuel cells. J. Mater. Chem..

[62] Kerres J., Atanasov V. (2015). Cross-linked PBI-based high-temperature membranes: Stability, conductivity and fuel cell performance. Int. J. Hydrogen Energy.

[63] Ozdemir Y., Ozkan N., Devrim Y. (2017). Fabrication and Characterization of Cross-linked Polybenzimidazole Based Membranes for High Temperature PEM Fuel Cells. Electrochim. Acta.

[64] Ossiander T., Perchthaler M., Heinzl C., Scheu C. (2014). Influence of thermal post-curing on the degradation of a cross-linked polybenzimidazole-based membrane for high temperature polymer electrolyte membrane fuel cells. J. Power Sources.

[65] Li X., Ma H., Wang P., Liu Z., Peng J., Hu W., Jiang Z., Liu B., Guiver M.D. (2020). Highly Conductive and Mechanically Stable Imidazole-Rich Cross-Linked Networks for High-Temperature Proton Exchange Membrane Fuel Cells. Chem. Mater..

[66] Hu M., Li T., Neelakandan S., Wang L., Chen Y. (2020). Cross-linked polybenzimidazoles containing hyperbranched cross-linkers and quaternary ammoniums as high-temperature proton exchange membranes: Enhanced stability and conductivity. J. Membr. Sci..

[67] Liu F., Wang S., Li J., Wang X., Yong Z., Cui Y., Liang D., Wang Z. (2021). Novel double cross-linked membrane based on poly (ionic liquid) and polybenzimidazole for high-temperature proton exchange membrane fuel cells. J. Power Sources.

[68] Liu F., Wang S., Wang D., Liu G., Cui Y., Liang D., Wang X., Yong Z., Wang Z. (2021). Multifunctional poly(ionic liquid)s cross-linked polybenzimidazole membrane with excellent long-term stability for high temperature-proton exchange membranes fuel cells. J. Power Sources.

[69] Tian X., Wang S., Li J., Liu F., Wang X., Chen H., Wang D., Ni H., Wang Z. (2019). Benzimidazole grafted polybenzimidazole cross-linked membranes with excellent PA stability for high-temperature proton exchange membrane applications. Appl. Surf. Sci..

[70] Wang P., Peng J., Yin B., Fu X., Wang L., Luo J.-L., Peng X. (2021). Phosphoric acid-doped polybenzimidazole with a leaf-like three-layer porous structure as a high-temperature proton exchange membrane for fuel cells. J. Mater. Chem. A.

[71] Liu F., Wang S., Chen H., Li J., Tian X., Wang X., Mao T., Xu J., Wang Z. (2018). Cross-Linkable Polymeric Ionic Liquid Improve Phosphoric Acid Retention and Long-Term Conductivity Stability in Polybenzimidazole Based PEMs. ACS. Sustain. Chem. Eng..

[72] Kim S.-K., Kim T.-H., Ko T., Lee J.-C. (2011). Cross-linked poly(2,5-benzimidazole) consisting of wholly aromatic groups for high-temperature PEM fuel cell applications. J. Membr. Sci..

[73] Harilal, Nayak R., Ghosh P.C., Jana T. (2020). Cross-Linked Polybenzimidazole Membrane for PEM Fuel Cells. ACS. Appl. Polym. Mater..

[74] Peng J., Fu X., Luo J., Liu Y., Wang L., Peng X. (2022). Constructing novel cross-linked polybenzimidazole network for high-performance high-temperature proton exchange membrane. J. Membr. Sci..

[75] Liu F., Wang S., Chen H., Li J., Wang X., Mao T., Wang Z. (2021). The impact of poly (ionic liquid) on the phosphoric acid stability of polybenzimidazole-base HT-PEMs. Renew. Energ..

[76] Arslan F., Chuluunbandi K., Freiberg A.T.S., Kormanyos A., Sit F., Cherevko S., Kerres J., Thiele S., Bohm T. (2021). Performance of Quaternized Polybenzimidazole-Cross-Linked Poly(vinylbenzyl chloride) Membranes in HT-PEMFCs. ACS. Appl. Mater. Inter..

[77] Wu Y., Liu X., Yang F., Zhou L.L., Yin B., Wang P., Wang L. (2021). Achieving high power density and excellent durability for high temperature proton exchange membrane fuel cells based on crosslinked branched polybenzimidazole and metal-organic frameworks. J. Membr. Sci..

[78] Guo H., Li Z., Lv Y., Pei H., Sun P., Zhang L., Cui W., Yin X., Hui H. (2021). Monolithic Macromolecule Membrane Based on Polybenzimidazole: Achieving High Proton Conductivity and Low Fuel Permeability through Multiple Cross-Linking. ACS Appl. Energ. Mater..

[79] Chen H., Wang S., Li J., Liu F., Tian X., Wang X., Mao T., Xu J., Wang Z. (2019). Novel cross-linked membranes based on polybenzimidazole and polymeric ionic liquid with improved proton conductivity for HT-PEMFC applications. J. Taiwan Inst. Chem. Eng..

[80] Choi S.-W., Park J.O., Pak C., Choi K.H., Lee J.-C., Chang H. (2013). Design and Synthesis of Cross-Linked Copolymer Membranes Based on Poly(benzoxazine) and Polybenzimidazole and Their Application to an Electrolyte Membrane for a High-Temperature PEM Fuel Cell. Polymers.

[81] Wang Y., Sun P., Li Z., Guo H., Pei H., Yin X. (2021). High performance polymer electrolyte membrane with efficient proton pathway over a wide humidity range and effective cross-linking network. React. Funct. Polym..

[82] Sun P., Li Z., Wang S., Yin X. (2018). Performance enhancement of polybenzimidazole based high temperature proton exchange membranes with multifunctional crosslinker and highly sulfonated polyaniline. J. Membr. Sci..

[83] Wang X., Wang S., Liu C., Li J., Liu F., Tian X., Chen H., Mao T., Xu J., Wang Z. (2018). Cage-like cross-linked membranes with excellent ionic liquid retention and elevated proton conductivity for HT-PEMFCs. Electrochim. Acta.

[84] Wang C., Li Z., Sun P., Pei H., Yin X. (2020). Preparation and Properties of Covalently Crosslinked Polybenzimidazole High Temperature Proton Exchange Membranes Doped with High Sulfonated Polyphosphazene. J. Electrochem. Soc..

[85] Li X., Ma H., Wang P., Liu Z., Peng J., Hu W., Jiang Z., Liu B. (2019). Construction of High-Performance, High-Temperature Proton Exchange Membranes through Incorporating SiO_2_ Nanoparticles into Novel Cross-linked Polybenzimidazole Networks. ACS Appl. Mater. Inter..

